# Supplementary material for: Transfer learning for mortality risk: A case study on the United Kingdom
Source: PLoS One. 2025 May 23;20(5):e0313378. doi: 10.1371/journal.pone.0313378 (PMC12101700; doi:10.1371/journal.pone.0313378)
Supplement: S1 Appendix — (PDF) [file pone.0313378.s001.pdf]

S1 Appendix. Methodology details of pretraining and specialization.

To pretrain the model we employ a popular ML technique: boosting machine. Boosting iteratively improves a regression model by fitting an additive expansion in a set of elementary basis functions, typically individual classifiers [1]. The model parameters undergo optimization through the training of Poisson boosted trees, with the objective of minimizing the negative log-likelihood associated with the Poisson distribution, serving as the designated loss function [2].

The pretrained model looks for patterns across countries and uses data from all countries to train it. It focuses on factors that can be compared between countries, like age. It excludes factors like occupation class, which can't be compared, but may have significant impact on mortality. With the local data, we calculate the specialized model. Each specialized model takes the output of the pretrained model from the first step and makes it more precise for that country. We find that incorporating local attributes during the latter phase of training offers optimal adaptability; this approach allows local nuances to be effectively integrated and, in cases where they are not applicable or transferable to the target country, they can be subsequently adjusted or mitigated. This way, we learn from the global experience on a larger dataset from different countries, while at the same time making it better for each country's specifics.

The predicted mortality rates from the global model are used to initialise the second local GBM models. The model works on the resulting residuals and optimises the second model with the local data set. The final predicted number of deaths is the result of multiplying the global model predictions, the local model predictions and the exposure. The following shows that the multiplication is justified by the boosting algorithm and the exponentiation by the log link of the Poisson distribution:

$$\begin{aligned}\mu_j &= \exp\left(\sum_{k=1}^{K} \theta_k \cdot u_k(X_j)\right) \\ &= \prod_{k=1}^{K} \exp\left(\theta_k \cdot u_k(X_j)\right) \overset{g\,:=\,\exp(\theta\,\cdot\,u(X))}{=} \underbrace{\prod_{k=1}^{T} g_k(X_j)}_{\text{global model } = \, q(.)} \cdot \underbrace{\prod_{l=T+1}^{K} g_l(X_j)}_{\text{local model } = \, h_j(.)}\end{aligned}$$

By splitting the modeling process into two steps, iterated from 1 to T and then from $T+1$ to K, the formulation cleanly segregates factors into local and global categories in sequentially. It also optimizes model performance for each market by tailoring the model to local patterns while allowing knowledge sharing across countries via the global model. Additionally, when onboarding a new country, we can choose to retain the existing global model and calculate a new local model for this new country.

References

1. Hastie T, Tibshirani R, Friedman JH. The elements of statistical learning: data mining, inference, and prediction. 2nd ed. Springer; 2009.

2. James G, Witten D, Hastie T, Tibshirani R, et al. An Introduction to Statistical Learning. Springer; 2013. 112th ed.
