# Supplementary material for: Transfer learning for mortality risk: A case study on the United Kingdom
Source: PLoS One. 2025 May 23;20(5):e0313378. doi: 10.1371/journal.pone.0313378 (PMC12101700; doi:10.1371/journal.pone.0313378)
Supplement: S2 Appendix — (PDF) [file pone.0313378.s002.pdf]

S2 Appendix. Lee-Carter model.

Lee Carter is a model for estimating mortality rates with the following assumptions [1]:

$$D_{a,t} \sim \text{Poisson}(E_{a,t} \cdot \mu_{a,t}), \quad \text{independently distributed} \tag{1}$$

$$\begin{aligned} &\eta_{a,t} = \alpha_a + \beta_a \kappa_t, \\ &\text{with} \ \ \log \mu_{a,t} = \eta_{a,t} \end{aligned} \tag{2}$$

Thus, fitting a Lee Carter model means basically to provide estimates for mortality rates using the two inputs exposure $E_{a,t}$ and death counts $D_{a,t}$ at age a and in year t for a given subpopulation. The expected number of deaths according to the Lee Carter fit can be calculated as $D^{LC}_{a,t} = E_{a,t} \cdot \mu^{LC}_{a,t}$.

References

1. Lee RD, Carter LR. Modeling and forecasting US mortality. Journal of the American Statistical Association. 1992;659–671.
