# Supplementary material for: Transfer learning for mortality risk: A case study on the United Kingdom
Source: PLoS One. 2025 May 23;20(5):e0313378. doi: 10.1371/journal.pone.0313378 (PMC12101700; doi:10.1371/journal.pone.0313378)
Supplement: S3 Appendix — (PDF) [file pone.0313378.s003.pdf]

S3 Appendix. Additional results of the drift model.

Insurers often require that certain relationships, such as between age and insurance risk, are monotonic, meaning they consistently increase or decrease without fluctuation [1, 2]. To address this, we introduce a method that employs GAM with a Poisson distribution and a log link function, integrated within our transfer learning framework. This approach uses smooth splines to model the complex, non-linear interplay between the age and gender as predictors and the predicted outcomes derived from transfer learning as target variable.

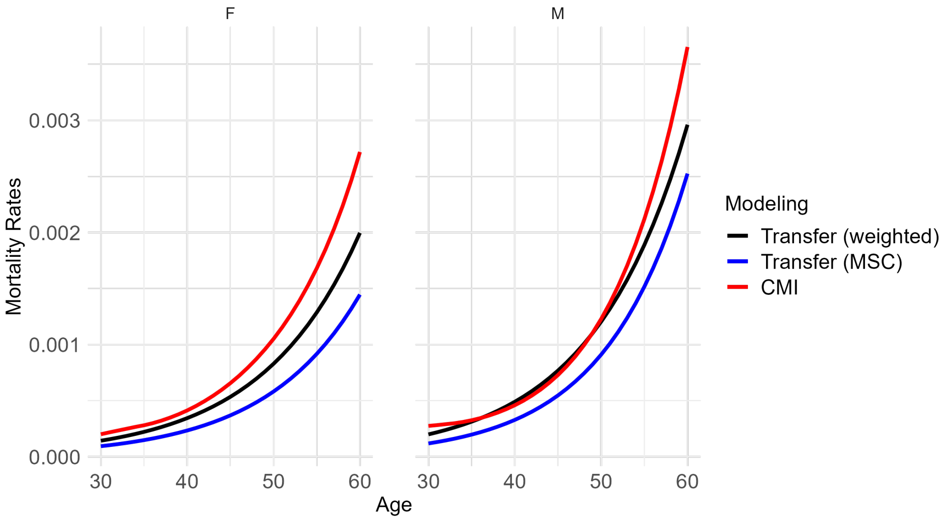

Fig 1. Comparison of UK mortality rates between Transfer Learning and CMI by age and gender. While transfer weighted by similarity score shows the above approach in black, the blue line shows the alternative of resampling only from the most similar country (MSC), which leads to a less accurate prediction. We present the smoothed version.

**Feature**	$\exp(\beta_{Feature})$	**Confidence Interval**
Intercept	0.6740739	[0.32352015, 1.4044739]
Age	0.9935284	[0.98699667, 1.0001034]
Gender: F	0.9077950	[0.85044196, 0.9690160]
Gender: M	1.1015706	[-, -]
Feature A: A1	0.6717810	[0.55795829, 0.8088233]
Feature A: A2	0.9270138	[0.78217372, 1.0986747]
Feature A: A3	1.0315687	[0.86443923, 1.2310107]
Feature A: A4	1.0683473	[0.88324160, 1.2922466]
Feature A: A5	1.1705879	[1.04346971, 1.3131921]
Feature A: A6	1.2447192	[-, -]
Feature B: B1	1.0720593	[0.93227045, 1.2328087]
Feature B: B2	1.0287833	[0.91022180, 1.1627881]
Feature B: B3	1.0025218	[0.86477463, 1.1622102]
Feature B: B4	0.9883311	[0.83156754, 1.1746470]
Feature B: B5	0.9150844	[-, -]
Feature C: C1	1.0157940	[0.89445867, 1.1535887]
Feature C: C2	0.9629419	[0.78382804, 1.1829855]
Feature C: C3	1.1107250	[0.94398197, 1.3069213]
Feature C: C4	0.8443653	[0.59959482, 1.1890577]
Feature C: C5	1.2260040	[0.96300772, 1.5608244]
Feature C: C6	1.0571062	[0.77361446, 1.4444837]
Feature C: C7	0.8410981	[-, -]
Feature D: D1	1.1292321	[0.77854251, 1.4917671]
Feature D: D2	0.7533651	[0.58801631, 1.1705793]
Feature D: D3	1.1754687	[-, -]
Feature E: E1	0.8269203	[0.61282725, 1.1158073]
Feature E: E2	0.8471899	[0.64778688, 1.1079735]
Feature E: E3	1.4274320	[-, -]
Feature F: F1	0.8417828	[0.62837821, 1.3410815]
Feature F: F2	1.3425524	[0.96974065, 1.7723960]
Feature F: F3	1.0298538	[0.82111288, 1.3075783]
Feature F: F4	0.9708001	[0.77889603, 1.4280173]
Feature F: F5	0.8252799	[0.66157520, 1.4755709]
Feature F: F6	1.5078144	[0.89935157, 1.7279372]
Feature F: F7	0.9327004	[0.66226747, 1.8818761]
Feature F: F8	0.8093660	[0.62934207, 1.3253971]
Feature F: F9	1.0081051	[0.65295776, 1.5564189]
Feature F: F10	0.9345903	[-, -]

Table 1. Estimated effects of the drift model with confidence intervals, where possible. The reference level is the grand mean.

References

1. Lim HB, Shyamalkumar ND. Incorporating industry stylized facts into mortality tables: Transfer learning with monotonicity constraints. 2024. Available from: `https://papers.ssrn.com/abstract=3964181`

2. American Academy of Actuaries, Society of Actuaries. 2015 Valuation Basic Table report. 2018. Available from: `https://www.soa.org/globalassets/assets/files/resources/experience-studies/2018/2015-vbt-report.pdf`
