# Supplementary material for: Transfer learning for mortality risk: A case study on the United Kingdom
Source: PLoS One. 2025 May 23;20(5):e0313378. doi: 10.1371/journal.pone.0313378 (PMC12101700; doi:10.1371/journal.pone.0313378)
Supplement: S4 Appendix — (PDF) [file pone.0313378.s004.pdf]

S4 Appendix. Bootstrap validation for confidence intervals.

Step 1: Resample the synthetic dataset

1. We treat the synthetic dataset X_M (generated using proportional resampling and noise augmentation) as the "population".
2. We use bootstrapping to create multiple resampled datasets. For each bootstrap iteration we:
   - Randomly sample rows from X_M **with replacement** until we have a dataset of the same size as X_M.
   - Record the row indices to ensure reproducibility.

Step 2: Predict mortality rates for each resampled dataset

1. Apply the pretrained global GBM model $q(X_M)$ and local specialized GBM model $h_j(X_M)$ to each bootstrap resample.
2. Calculate the mortality rates $\hat{\mu}_{M,b}$ for bootstrap iteration b using the combined model output:
$$\hat{\mu}_{M,b} = \sum_{j=1}^{K} q(X_{Mj}) \cdot h_j(X_{Mj})$$
3. Store the predicted mortality rates for each bootstrap iteration.

Step 3: Compute confidence intervals

1. For each demographic segment (e.g., age-gender group), we aggregate the predicted mortality rates from all bootstrap iterations B to calculate:
   - Mean predicted mortality rate $\bar{\mu}_M$ across bootstraps.
   - Confidence intervals using the percentiles of the bootstrap predictions $\hat{\mu}_{M,1}, \hat{\mu}_{M,2}, \ldots, \hat{\mu}_{M,B}$. For 95% confidence intervals:
$$\text{Lower Bound} = \text{Percentile}_{2.5}(\{\hat{\mu}_{M,b}\})$$
$$\text{Upper Bound} = \text{Percentile}_{97.5}(\{\hat{\mu}_{M,b}\})$$
