# Supplementary material for: Transfer learning for mortality risk: A case study on the United Kingdom
Source: PLoS One. 2025 May 23;20(5):e0313378. doi: 10.1371/journal.pone.0313378 (PMC12101700; doi:10.1371/journal.pone.0313378)
Supplement: S5 Appendix — (PDF) [file pone.0313378.s005.pdf]

S5 Appendix. Workflow for synthetic data generation.

We provide a stepwise description of the workflow used to generate the synthetic dataset for mortality prediction in the target country based on data from other source countries.

1. Build country similarity index

- **Input:** Demographic, economic, and health-related variables for both target and source countries.
- **Steps:**
  1. Preprocess variables by centralizing and scaling to standardize the data.
  2. Apply Principal Component Analysis (PCA) to reduce dimensionality while retaining >95% variance.
  3. Compute Manhattan distances between target and all other countries for the reduced variables, resulting in 8 distance measures.
  4. Transform Manhattan distances into similarity scores using the Generalized Radial Basis Function (RBF) kernel to normalize similarity scores to the range $(0, 1]$.

2. Maximize the final similarity score

- Combine similarity scores across multiple dimensions to maximize alignment with the target by:
  - Weighting each dimension based on its relative importance (pre-defined).
  - Weighting the source countries according to the similarity score.

3. Calculate overall population mortality rates from HMD

- **Input:** Mortality data from each source country.
- **Steps:**
  1. Extract overall population mortality rates from source countries for each demographic subgroup (e.g., age, gender).
  2. Populate the respective feature column in the dataset for the target country by incorporating and replacing it by the target country's overall population mortality rates from HMD.

4. Construct the synthetic dataset

- **Input:** Demographic distribution for the target country.
- **Steps:**
  1. **Receive or borrow demographic assumptions:**
     - Use age-gender distribution for target country if available.
     - Otherwise, borrow the distribution from the most similar country.

2. **Resample from all countries, drawing according to this distribution and proportionally to the similarity score for each country and combine.**
3. **Perform data augmentation with noise according to the description in Methodology section. The higher the similarity score, the lower the noise parameter.**

5. Predict

- **Input:** Dataset from Step 4.
- **Steps:**
  1. Use the dataset created in Step 4 for mortality predictions of target country.
  2. Refit the global pretrained model for the entire dataset.
  3. Combine predictions by multiplying the outputs from the global model with those from each country's localized model.

Output

- Synthetic dataset enriched with real HMD data of target country.
- Predicted mortality rates for target country based on the other countries' data.
